# Near-Earth plasma sheet boundary dynamics during substorm dipolarization

**DOI:** 10.1186/s40623-017-0707-2

**Published:** 2017-09-18

**Authors:** Rumi Nakamura, Tsugunobu Nagai, Joachim Birn, Victor A. Sergeev, Olivier Le Contel, Ali Varsani, Wolfgang Baumjohann, Takuma Nakamura, Sergey Apatenkov, Anton Artemyev, Robert E. Ergun, Stephen A. Fuselier, Daniel J. Gershman, Barbara J. Giles, Yuri V. Khotyaintsev, Per-Arne Lindqvist, Werner Magnes, Barry Mauk, Christopher T. Russell, Howard J. Singer, Julia Stawarz, Robert J. Strangeway, Brian Anderson, Ken R. Bromund, David Fischer, Laurence Kepko, Guan Le, Ferdinand Plaschke, James A. Slavin, Ian Cohen, Allison Jaynes, Drew L. Turner

**Affiliations:** 1 Space Research Institute, Austrian Academy of Sciences, Graz, Austria; 2 Tokyo Institute of Technology, Tokyo, Japan; 3 Space Science Institute, Boulder, CO USA; 4 St. Petersburg State University, St. Petersburg, Russia; 5 Laboratoire de Physique des Plasmas, CNRS/Ecole polytechnique/UPMC Univ Paris 06/Univ. Paris-Sud/Observatoire de Paris, Paris, France; 6 University of California, Los Angeles, CA USA; 7 LASP, University of Colorado, Boulder, CO USA; 8 Southwest Research Institute, San Antonio, TX USA; 9 NASA, GSFC, Greenbelt, MD USA; 10 Swedish Institute of Space Physics, Uppsala, Sweden; 11 Royal Institute of Technology, Stockholm, Sweden; 12 Applied Physics Laboratory, Johns Hopkins University, Laurel, MD USA; 13 NOAA Space Weather Prediction Center, Boulder, CO USA; 14 Department of Physics, Imperial College London, London, UK; 15 Department of Climate and Space Sciences and Engineering, University of Michigan, Ann Arbor, MI USA; 16 Space Sciences Department, Aerospace Corporation, Los Angeles, CA USA

**Keywords:** Substorm, Dipolarization, Plasma sheet boundary layer, Field-aligned current

## Abstract

We report on the large-scale evolution of dipolarization in the near-Earth plasma sheet during an intense (AL ~ −1000 nT) substorm on August 10, 2016, when multiple spacecraft at radial distances between 4 and 15 *R*_E_ were present in the night-side magnetosphere. This global dipolarization consisted of multiple short-timescale (a couple of minutes) *B*_*z*_ disturbances detected by spacecraft distributed over 9 MLT, consistent with the large-scale substorm current wedge observed by ground-based magnetometers. The four spacecraft of the Magnetospheric Multiscale were located in the southern hemisphere plasma sheet and observed fast flow disturbances associated with this dipolarization. The high-time-resolution measurements from MMS enable us to detect the rapid motion of the field structures and flow disturbances separately. A distinct pattern of the flow and field disturbance near the plasma boundaries was found. We suggest that a vortex motion created around the localized flows resulted in another field-aligned current system at the off-equatorial side of the BBF-associated R1/R2 systems, as was predicted by the MHD simulation of a localized reconnection jet. The observations by GOES and Geotail, which were located in the opposite hemisphere and local time, support this view. We demonstrate that the processes of both Earthward flow braking and of accumulated magnetic flux evolving tailward also control the dynamics in the boundary region of the near-Earth plasma sheet.

**Electronic supplementary material:**

The online version of this article (doi:10.1186/s40623-017-0707-2) contains supplementary material, which is available to authorized users.

**Graphical Abstract:**
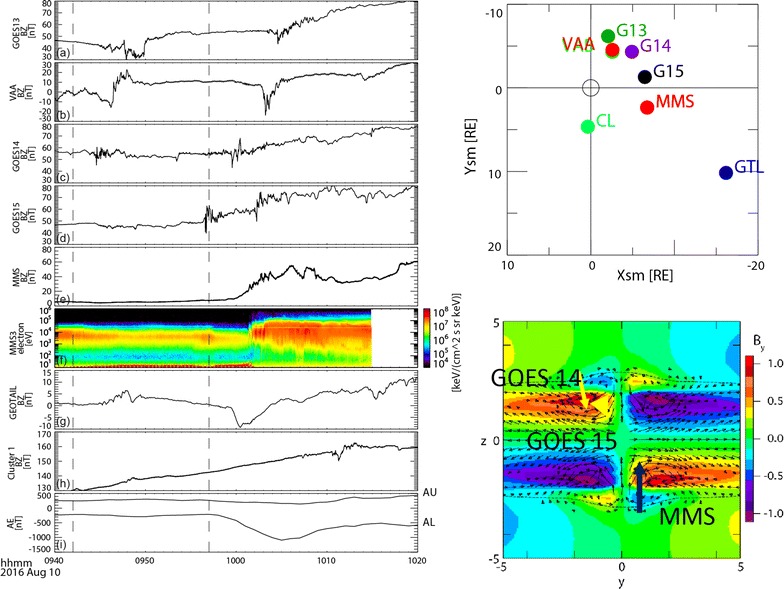
Multispacecraft observations of dipolarization (*left panel*). Magnetic field component normal to the current sheet (BZ) observed in the night side magnetosphere are plotted from post-midnight to premidnight region: *a* GOES 13, *b* Van Allen Probe-A, *c* GOES 14, *d* GOES 15, *e* MMS3, *g* Geotail, *h* Cluster 1, together with *f* a combined product of energy spectra of electrons from MMS1 and MMS3 and *i* auroral electrojet indices. Spacecraft location in the GSM X-Y plane (*upper right panel*). Colorcoded By disturbances around the reconnection jets from the MHD simulation of the reconnection by Birn and Hesse (1996) (*lower right panel*). MMS and GOES 14-15 observed disturbances similar to those at the location indicated by arrows

## Introduction

Substorms, the most dynamic process in the Earth’s magnetotail, result in large-scale reconfiguration of the magnetotail current sheet and enhanced coupling between the magnetosphere and ionosphere, driven by near-Earth magnetotail reconnection and other current sheet instabilities. The main magnetospheric signatures of substorms are thinning and expansion of the plasma sheet, magnetic field dipolarization, enhanced occurrence of fast plasma flows, energetic particle injection, and intensified field-aligned currents (FACs).

The main driver of the near-Earth magnetotail disturbances is the localized fast plasma jets, called bursty bulk flows (BBFs), jetting Earthward and interacting with the ambient plasma to form a thin front layer called the dipolarization front. During the substorm expansion phase, major energy dissipation and current disruption (unloading) processes take place in the flow-braking region, i.e., the region where the probability of detecting Earthward BBFs significantly drops (e.g., Sergeev et al. 2012, and references therein). From the decrease in occurrence frequency of observing rapid flux transport rate (enhanced dawn-to-dusk electric field, *E*_*Y*_), it was suggested that flow braking takes place between 10 and 15 *R*_*E*_ (Schödel et al. 2001). The statistical average of the *E*_*Y*_ in the dipolarization front, however, is larger closer to the Earth (Tu et al. 2000; Liu et al. 2014; Schmid et al. 2016), which indicates that the flux transport rate itself increases before the flows brake. This inference is consistent with the large dawn-to-dusk electric field obtained in MHD simulations due to the induced electric field because the magnetic flux piles up in the flow-braking region (Birn and Hesse 1996; Birn et al. 2011). It should be noted, however, that a departure from the frozen-in condition has also been reported in this region (e.g., Lui 2013). Furthermore, because of the localized and transient natures of BBFs, the details of individual flow braking and the relationships between flow braking and overall substorm dipolarization remain to be elucidated.

A large-scale current system, called the substorm current wedge (SCW), develops during the substorm expansion phase. This current system was first considered to explain ground-based mid-latitude magnetogram data (McPherron et al. 1973). It consists mainly of a net current along the magnetic field toward the magnetosphere outward from the ionosphere (upward FAC) at the western edge and into the ionosphere at the eastern edge of the auroral activity, connected by a westward horizontal current in the ionosphere. This current system also explained the magnetic perturbation of the geosynchronous orbit (Nagai 1982). A similar field-aligned pattern was also identified during small-scale substorms/pseudobreakups (Nakamura et al. 2001; Palin et al. 2015) and during auroral streamers and BBFs (e.g., Henderson et al. 1998; Nakamura et al. 2001). The current system described above has a sense of night-side “Region 1” (R1) (Iijima and Potemra 1978), which represents a statistical pattern of the FAC in the high-latitude ionosphere detected by low-Earth orbit spacecraft. Another type of FAC called “Region 2” (R2), with the sense opposite to that of R1 (upward at eastern edge, downward at western edge), was detected in low-Earth orbit observations at the equatorward side of R1. A pair of FACs with the R2 sense was also found in the dipolarization front/dipolar flux bundle ahead of the R1 FAC (Liu et al. 2013). In this region, R1 typically dominates R2 by more than a factor of 2, as shown based on empirical modeling of the SCW (Sergeev et al. 2011) and in MHD simulation (Birn and Hesse 2014). The statistical pattern reported by Iijima and Potemra (1978) showed that in the near-midnight region (i.e., near the demarcation line of the current system), the post-midnight R1 (downward) current is located on the poleward side of the pre-midnight R1 (upward) current. A downward FAC has also been observed in the ionosphere at the poleward boundary of the auroral bulge and is called “Region 0” (R0) (Fujii et al. 1994; Gjerloev and Hoffman 2002). Nagai et al. (1987) reported magnetic disturbances near the geosynchronous orbit caused by a downward current located outside the upward current near the center of the SCW during a substorm event. However, the link of R0 between the ionosphere and the magnetosphere is not well understood. These observations indicate that the actual magnetosphere–ionosphere coupling processes are more complex, although the net effect seen in ground-based mid-latitude magnetic field disturbances may show disturbance of a one-loop wedge-like system. Details of the magnetospheric and ionospheric observations related to SCW are reviewed by Kepko et al. (2015).

BBF and dipolarization fronts in the central plasma sheet (CPS) produce transient ion beams accompanied by magnetic field disturbances in the near-Earth plasma sheet boundary layer (PSBL) (Zhou et al. 2012). Therefore, the disturbances in the rapid flux transport process can take place across the entire near-Earth plasma sheet. Although fast Earthward flows perpendicular to the field are most clearly detected in the CPS, 3D MHD and particle-in-cell simulations have shown that the local current diversion producing FAC is more pronounced in the shear layers above and below the neutral sheet rather than close to the neutral sheet (e.g., Birn and Hesse 1996; Pritchett et al. 2014).

On August 10, 2016, the Magnetospheric Multiscale (MMS) crossed the near-Earth tail region when an intense substorm commenced at 09:57 UT. The fleet of spacecraft (MMS, Geotail, GOES 13–15, Van Allen Probes, Cluster) were distributed in the night-side magnetosphere and detected the substorm dipolarization signatures. For this study, we have investigated both the large-scale evolution of this dipolarization and the detailed structures of the boundaries relevant to this dynamic FAC system.

## Observations

**Fig. 1:**
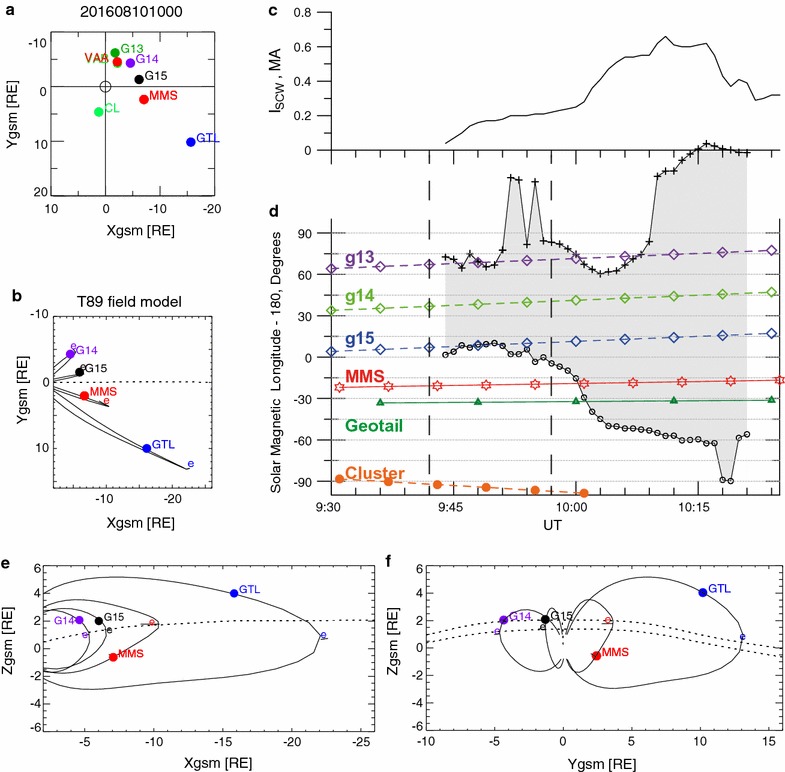
Spacecraft location and magnetic field model field lines from the T89 model at 10:00 UT on August 10, 2016, and location of SCW. **a** Location of VAA and VAB (*red* and *green*); GOES 13, 14, and 15 (*green*, *purple*, and *black*); MMS (*red*), Cluster 1 (*light green*); Geotail (*dark blue*) and **b** MMS, GOES 14 and 15, Geotail, and traced field lines using the T89 model projected in a GSM *X*–*Y* plane. **c** Total current of the SCW and **d** magnetic local time of the SCW and spacecraft obtained using the SCW model (Sergeev et al. 2011). Location of MMS, GOES 14, 15, Geotail, and traced field lines using T89 model projected (**e**) in the GSM *X*–*Z* plane and **f** in the GSM *Y*–*Z* plane. The location of the equator is denoted by “**e**”. The *dotted line* in **e** indicates the location of the equatorial plane in the midnight meridian (*Y* = 0), whereas those in **f** show the equatorial plane at *X* = −10 *R*_*E*_ (*upper curve*) and *X* = −7 *R*_*E*_ (*lower curve*)

An intense substorm with AE exceeding 1000 nT took place on August 10, 2016, with two main positive bay onsets around 09:42 UT and 09:57 UT. Despite the intense electrojet at the auroral latitude, no Dst enhancement accompanied this event, which indicates that no pronounced enhancement in the ring current took place. The interplanetary magnetic field was weakly southward (−2 to −3 nT) for an interval of about 90 min before the onset of substorm expansion. During this substorm interval, the two Van Allen Probes and the Geostationary Operational Environmental Satellites (GOES) 13–15 spacecraft were located in the post-midnight sector, while the MMS, GEOTAIL, and Cluster were located between the pre-midnight and dusk sectors (Fig. 1a, b). A SCW with a maximum intensity of about 0.6 MA (Fig. 1c), deduced using the SCW model (Sergeev et al. 2011), was centered at post-midnight for onset at 09:42 UT and expanded toward the pre-midnight region for the 09:57 UT onset, and thus extended to local time sectors where the MMS was located (Fig. 1d).

**Fig. 2:**
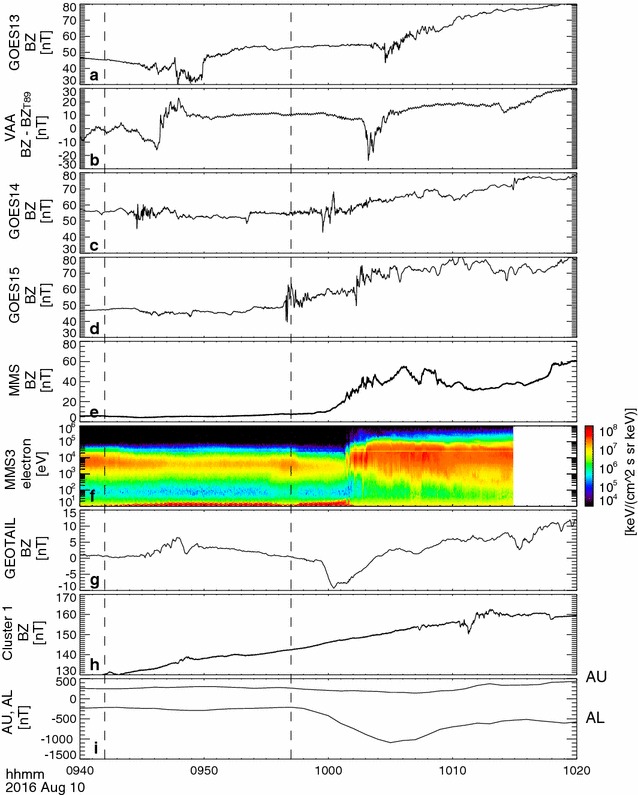
Multispacecraft observations of dipolarization. Magnetic field component normalized to the current sheet (*B*_*Z*_) observed in the night-side magnetosphere is plotted from the post-midnight to pre-midnight regions: **a** GOES 13, **b** Van Allen Probe-A, **c** GOES 14, **d** GOES 15, **e** MMS3, **g** Geotail, **h** Cluster 1, together with **f** a combined product of energy spectra of electrons from MMS1 and MMS3 and **i** auroral electrojet indices. Electron spectra for energy lower/higher than 25 keV are plotted using MMS3 FPI/MMS1 EIS data. For VAA traversing *B*_*Z*_, the difference from the model magnetic field (T89) is plotted. The* vertical line* indicates 09:42 and 09:57 UT, which are the positive bay onset times

Figure 2 shows the Bz (northward) component of the magnetic field from GOES 13, 14, and 15 (Singer et al. 1996), Van Allen Probe-A (Kletzing et al. 2013), MMS 3 (Russell et al. 2016), Geotail (Kokubun et al. 1994), and Cluster 1 (Balogh et al. 2001) and electron energy spectra from the MMS Energetic Ion Spectrometer (EIS) (Mauk et al. 2014) and the Fast Plasma Instruments (FPI) (Pollock et al. 2016) instrument together with the AU and AL indices. The electron energy spectra (panel f) are a combined data product from the MMS1 EIS instrument for energy >25 keV and from the MMS3 FPI instrument for energy <25 keV. To enhance the visibility of the high-energy portion, the EIS electron energy flux is increased by a factor of 2.75 in the panel. Here, we have used the solar magnetic coordinates (SM) for all the above-mentioned spacecraft except for Geotail, for which the geomagnetic solar magnetospheric coordinates (GSM) were used, because only Geotail was located behind the typical hinging distance in a more tail-like region (see Fig. 1e). For Van Allen Probe-A (VAA), which quickly traversed different L shells, the difference between observed field and the model magnetic field (T89) (Tsyganenko 1989) was plotted. As expected from the SCW distribution (Fig. 1d), the 09:42 UT onset is associated with the Bz disturbance, mainly in the post-midnight sector. The 09:57 UT onset, on the other hand, was first observed at GOES 15 along with some enhancement in the electron flux at MMS, followed by successive Bz disturbances detected at Geotail, MMS, GOES 13–15 and VAA, hence, expanding/propagating duskward and dawnward and covering a region of the SCW extending over the 9 MLT wide region (Fig. 1d). Bz disturbances also took place after 10:10 UT in all the spacecraft intermittently when the modeled SCW region covers the entire night-side local time.

**Fig. 3:**
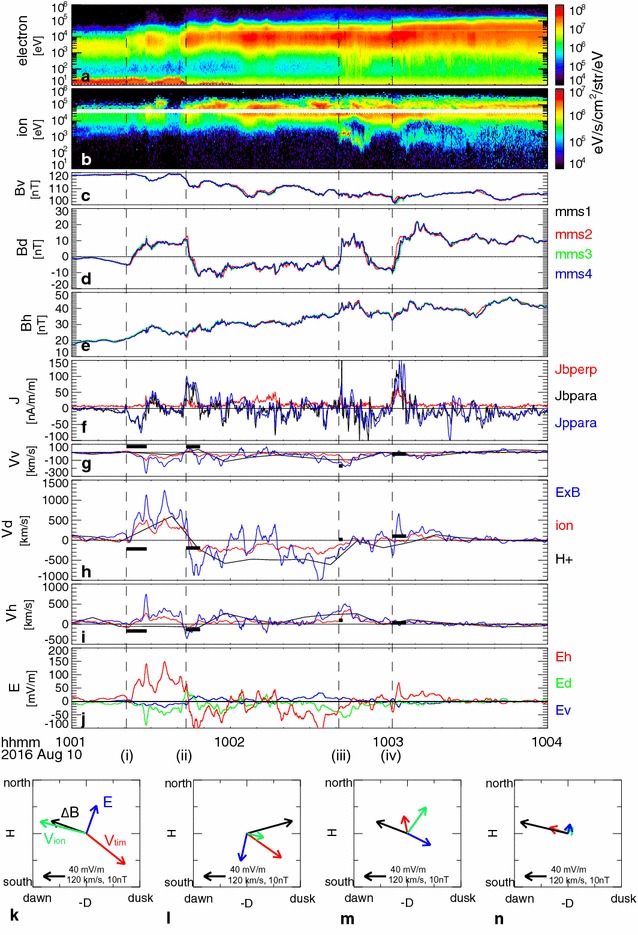
MMS field and particle observations during dipolarization. Energy spectra from **a** electrons and **b** ions obtained from the EIS and FPI instruments. Electron spectra for energy lower/higher than 25 keV are plotted using FPI and EIS data. Ion spectra for energy higher/lower than 45/30 keV are plotted using EIS/FPI data. **c** *V*, **d** *D*, and **e** *H* components of the magnetic fields from the four MMS spacecraft. **f** *P* parallel (*black*) and perpendicular (*red*) components of the currents determined using the curlometer method and parallel current calculated using FPI ion and electron moments (*blue curve*). **g** *V*, **h** *D*, and **i** *H* components of the plasma flows perpendicular to the magnetic field and *E* × *B* drifts. The *blue curves* in **g**–**i** correspond to *E* × *B* drift obtained from EDP, whereas the *red curves* are the ion velocity from FPI, and the *black curves* show the proton velocity from HPCA. **j** *V*, *D*, and *H* components of the electric fields. The *vertical dashed lines* show the start of the crossing times of the main current layers associated with the dipolarization: (*i*) 10:01:22, (*ii*) 10:01:43, (*iii*) 10:02:4, and 1 (*iv*) 10:03:01. The *horizontal black bars* in **g**–**i** present the timing velocity. This velocity is determined from the time delays between the four spacecraft in the *B*_*D*_ traces during the time interval represented, and is indicated by the lengths of the *horizontal bars*. The four* bottom panels* (**k**–**n**) show the average ion flows perpendicular to the magnetic field (*green*) and electric field (*dark blue*) during the four current sheet crossings (*i*–*iv*). The magnetic field disturbance vector (difference between the end and the start time of the crossing) is shown in *black*, and the timing velocity vector is in *red*

Particle and field observations at MMS during the main dipolarization events (between 10:01 and 10:04 UT) are shown in Fig. 3. Panel a shows the energy spectra of electrons with the same format as Fig. 2f. Panel b shows ion energy spectra from MMS3 plotted using proton data from EIS for higher energy (>45 keV) and ion data from FPI for lower energy (<30 keV). For FPI ion data, background noise caused by energetic electrons was subtracted. To enhance the visibility of the high-energy part, the EIS proton energy flux was multiplied by 5. MMS was located in the outer plasma sheet until around 10:01:30 UT, but entered a hotter plasma sheet region during the dipolarization event. This transition from outer to center plasma sheet can also be seen in the magnetic field data (Fig. 3c–e) plotted in *VDH* coordinates, which better represent the magnetic disturbance at different local times in a dipolar configuration. Here, *H* is along the geomagnetic dipole axis and positive northward, and is therefore the same as the *Z* component in the *SM* coordinate system. *D* is perpendicular to the radial direction, *R*, and *H*, i.e., *H* × *R*, and is positive eastward. *V* denotes the radially outward direction, which corresponds approximately to the background field direction, and is closing the right-hand coordinate system. The overall changes from the tail-like configuration to a more dipolar configuration, as expected from the plasma profile, can well be identified based on the decrease in *B*_*V*_ (panel c) as well as increase in *B*_*H*_ (panel e).

The dipolarization (enhancement in *B*_*H*_) consists of multiple short-timescale enhancements. These enhancements are accompanied by large changes in *B*_*D*_, which indicate a crossing of the FAC sheets, as confirmed based on the FAC calculated using the curlometer method (Chanteur and Harvey 1998) and the four MMS spacecraft (black curve in panel f). Parallel currents calculated using particle data from the FPI onboard MMS3 (blue curve in panel f) coincide well with the curlometer current, which indicates that the spatial scales are sufficiently large relative to the inter-spacecraft distances, typically around 50 km. There are four main rapid crossings of intense FAC layers (*B*_*D*_ change), all accompanied by dipolarization fronts (*B*_*H*_ enhancements), as indicated by dashed lines. Each corresponds to the start time of a sharp *B*_*D*_ change at: (1) 10:01:22, (2) 10:01:43, (3) 10:02:41, and (4) 10:03:01. The direction of the FAC for event (1) was anti-parallel to the field direction; that is, currents flowing into the ionosphere. The FACs for the other three events, (2)–(4), were directed parallel to the field direction, which means the currents flowed out from the ionosphere (upward FAC). Because the MMS is located in the dusk part of the current wedge, the event (1) was an R2-sense current, whereas the others were R1 sense currents.

Figure 3g–i shows plasma flows perpendicular to the magnetic field and *E* × *B* drifts, and Fig. 3j shows the electric field (Lindqvist et al. 2014). Here, the electric field (panel j and blue traces in panels g–i) and FPI ion velocity data (red traces in panels g–i) are averaged to 1-Hz resolution (i.e., about the ion-gyro frequency). Proton velocity data obtained by the Hot Plasma Composition Analyzer (HPCA) instrument (Young et al. 2014) are shown in 10-s resolution as black curves in panels g–i. The transient dipolarizations and the crossings of the current layers are associated with enhancements in the equatorward/Earthward plasma flows and in the dawn-to-dusk electric field (negative *E*_*D*_), as represented in Fig. 3g, i, j, except for the first part of event (2), when *E*_*D*_ changed from dawnward to duskward and flow changed from outward/tailward to inward/Earthward. The intense flux extending to higher energy in the ion spectra suggests that the obtained ion velocity is likely underestimated, particularly after the plasma sheet proper is entered. Nonetheless, the overall traces show similar changes among the three velocity estimates. All four FAC events were accompanied by a strong dawn-to-dusk electric field (negative *E*_*D*_) with a magnitude exceeding several 10 s mV/m. This electric field strength is comparable or slightly stronger than the upper quartile value of the electric field of dipolarization flux bundle events in the CPS in the 6 *R*_*E*_ region obtained from a statistical study (Liu et al. 2014). However, the largest changes take place in the east–west flows (i.e., dawn–dusk flows), *V*_*D*_, and the north–south electric field *E*_*H*_, except for in event (3). In particular, events (1) and (2) were associated with intense dawnward flow and reversal to duskward flows, respectively. One candidate to explain these strong dawn–dusk flows may be deflection of the BBFs in the flow-braking regions. However, in such cases, duskward flows such as that observed in event (2) are a more natural flow direction considering the local time of MMS relative to SCW rather than dawnward flows as observed in event (1).

The four MMS spacecraft were separated by about 50 km during this event and detected quite similar traces in the magnetic fields, except with some time shifts (Fig. 3c–e). Such a profile is ideal to infer the motion of the magnetic structures using the timing method. The obtained velocity of the magnetic structure, which we call “timing velocity,” *V*_tim_, is plotted with horizontal bars in panels g–i. The timing velocity is inferred from the time difference between the spacecraft, which is determined by cross-correlating the *B*_*D*_ components between different spacecraft during the time interval indicated as the horizontal length of the bars in panels g–i (horizontal black bars in Fig. 3). The relationships between *V*_tim_ (red), the average ion velocity perpendicular to the magnetic field, *V*_ion_ (green), and the electric field, *E* (blue), during the four events are given in the panels k–n in the H–D plane. It can be seen that *V*_tim_ during the FAC events (1) and (2) was directed southward/westward (~duskward), whereas during events (3) and (4), it was directed northward/dawnward. Note that these FACs (flowing mainly along *V*) were produced by magnetic disturbances predominantly along *D*, as indicated by the black arrow, Δ*B*, which is the difference in *B* vectors between the start and end time of each FAC-crossing event. Therefore, the timing velocity along *H* represents the motion of the current sheet. The outward (southward) motion of the current sheet in events (1) and (2) is likely associated with plasma sheet expansion. The motion of both the plasma (*V*_ion_) and the boundary (*V*_tim_) is equatorward for events (3) and (4), as is often the case for dipolarization fronts of enhanced Earthward fast flows. These different motions of the boundary change the polarity of the FAC direction with respect to the change in *B*_*D*_. Because the main magnetic disturbances are caused by the FAC, Δ*B*_*D*_/*V*_tim*H*_, they will provide the polarity of FAC. This value is negative for event (1) and positive for the other three events, consistent with the curlometer current. For events (1)–(3), enhancements in the northward/equatorward motion of plasma were detected with respect to the boundary, as expected in the enhanced dawn-to-dusk convection electric field, whereas the FAC event (4) took place after the convection electric field enhancement. If we estimate the spatial scale of the four current sheets from *V*_tim*H*_ multiplied by the crossing times, we obtain the thicknesses of the current sheet for each event: (1) 1310 km, (2) 710 km, (3) 140, and (4) 170 km.

**Fig. 4:**
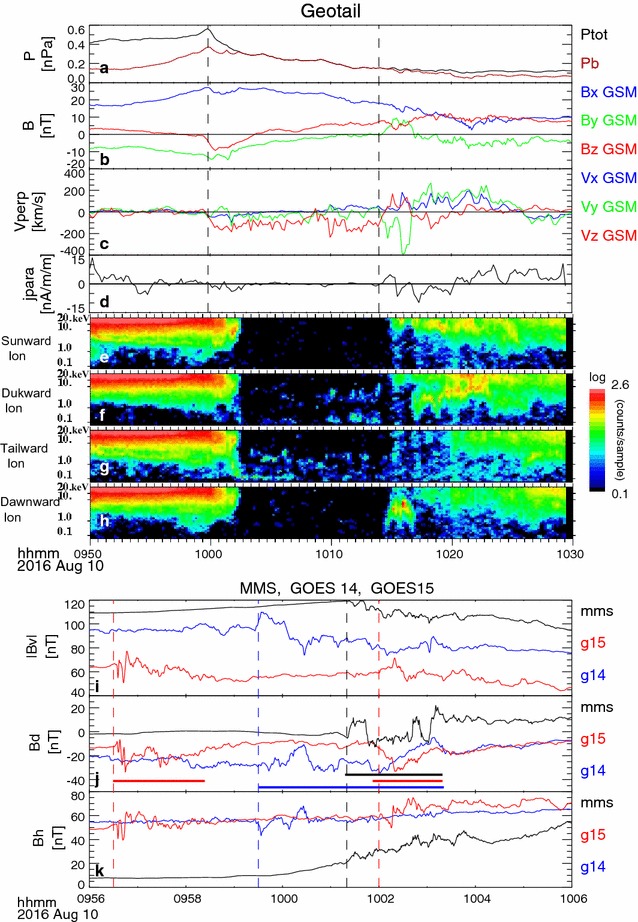
Geotail particle and field observations during thinning and expansion of the plasma sheet (*upper panels*) and GOES 14–15 and the MMS magnetic field observations during the dipolarization events (*lower panels*). **a** Total pressure (*black*) and magnetic pressure (*brown*), **b** *X*, *Y*, and *Z* components of the magnetic fields, **c** ion flows perpendicular to the magnetic field, and **d** parallel current determined from ion and electron moment data and energy spectra of ions streaming sunward (**e**), duskward (**f**), tailward (**g**), and dawnward (**h**) as observed by Geotail between 09:50 and 10:30 UT. The *dashed lines* in **a**–**h** show the beginnings of the disturbances associated with the exit and reentry of the plasma sheet. **i** Absolute value of the *V*, **j** *D* and **k** *H* components of the magnetic field from MMS 3 (*black*) GOES 15 (*red*) and GOES 14 (*blue*) between 09:56 and 10:06 UT. The *dashed lines* in **i**–**k** show the start of the rapid dipolarization fronts for each spacecraft. The *horizontal bars* highlight the disturbances in the *D* components associated with dipolarization (discussed in greater detail in the text)

Figure 4a–h shows magnetic field and plasma data from the low-energy particle instrument (LEP) (Mukai et al. 1994) from Geotail observations between 09:50 and 10:30 UT. The MLT of Geotail was 22 MLT based on the spacecraft location (Fig. 1d), which corresponds to 21 MLT for its ionospheric foot point, taking into account the stretched tail-like configuration. Geotail was located west of the SCW during the 09:57 UT onset, but the westward expansion of the current wedge region then crossed the Geotail local time between 10:00 and 10:05 UT. At 9:59:50, there was a ~1 min long ~10% compression in the Bx centered on the inflection point of a slightly positive, then negative Bz variation with similar duration. This observation suggests a signature of a tailward-moving traveling compression region (TCR), which has been shown to be caused by the draping of the lobe magnetic field flux tubes about plasmoid-type flux ropes ejected down the tail by the plasma exhaust from *x*-lines in the plasma sheet (Slavin et al. 1984). Enhanced southward flows, corresponding to the dawn-to-dusk electric field of up to 2.5 mV/m, continued afterward, accompanied by a decrease in pressure, which indicates unloading and exiting to the lobe. Such signatures have been reported as the current sheet thinning associated with the enhanced flux transport rate due to activation of near-Earth reconnection tailward of the spacecraft (Sergeev et al. 2008). Therefore, Geotail was likely close to the reconnection region and then detected its tailward progression. Geotail then entered the plasma sheet, accompanied by enhancement in *B*_*Z*_ from 10:14 UT. Positive/negative *B*_*Y*_ and negative/positive *V*_*Y*_ in the lobe/plasma sheet side were observed during the crossing. Note that the change in the dawnward to duskward motion of the plasma occurred mainly in the lower energy (~few keV) population, whereas the Earthward high-energy (~10 to 20 keV) ion beams were deflected duskward. This pattern is similar to the FAC events (1) and (2) observed by MMS, associated with outward motion of the current sheet. The FAC deduced from the particle spectra also shows a consistent pattern; that is, a downward current lobe-side and upward current at the plasma sheet side. The parallel current was calculated from the ion and electron moments, with the 12-s moment data averaged over five points to reduce noise. Note that in the plasma sheet region (after around 10:20 UT), when the amplitude of fluctuation was too large relative to the background field, determination of parallel current becomes less reliable.

Magnetic field data from GOES 14, 15, and MMS between 09:56 and 10:06 UT are shown in Fig. 4i–k. From the SCW location (Fig. 1d), it is expected that GOES 14 is located at the dawnward side of the current wedge center, whereas GOES 15 is near the duskside of the current wedge center. In such cases, the expected perturbation in *B*_*D*_ at geosynchronous altitude in the northern hemisphere would be positive perturbation for GOES 15 and negative for GOES 14, assuming the SCW is located tailward of the spacecraft. However, the pattern was the opposite, as highlighted by the red and blue bars in Fig. 4j. During this onset, another peak in the *D* appeared in the pre-midnight, overlapped with a broader disturbance, which is visible in Additional file 1: Figure S1. This finding suggests an asymmetric disturbance caused by a localized pre-midnight SCW in addition to the broader disturbance modeled as one SCW (Fig. 1d; Additional file 1: S1). The high-latitude magnetograms (Additional file 1: Figure S2) also show strong westward electrojet, which was concentrated in the pre-midnight near the footpoint of MMS, and support this view. GOES 14 and 15 were therefore more likely located dawnside of this localized current wedge developed in the pre-midnight region. Furthermore, the relatively low-latitude location of the strong westward electrojet, as well as the possible *X*-line location near Earth, suggests that SCW was located near the geosynchronous orbit. The difference between GOES 14 and GOES 15 can then be interpreted as caused by the difference in the distance from the center of the current sheet. That is, GOES 14/15 observed the downward FAC, corresponding to the dawnside part of the localized intense SCW located equatorward/poleward of the spacecraft. The profile of the *B*_*D*_ perturbations from GOES 14 therefore resembles that of MMS because of the opposite hemisphere and opposite local time relative to the current system compared with MMS.

## Discussion

A large-scale dipolarization during an intense substorm expansion phase was monitored by a fleet of spacecraft in the near-Earth magnetotail on August 10, 2016. A SCW developed with main activations at 09:42 and 09:57 UT, according to ground-based observations. While the SCW at 09:42 UT was located mainly in the post-midnight, the SCW at 09:57 UT developed in a wider region, including the pre-midnight where MMS and Geotail were located. As expected, at the western edge of the SCW, the main magnetic disturbances at MMS were caused by intense upward FACs with large amplitudes exceeding 20 nT. This large value indicates that the total current value of ~0.25 MA around 10:01 UT observed on the ground (Fig. 1c) can be achieved with a FAC sheet with an azimuthal width of about 2.5 *R*_*E*_ at maximum. The corresponding current wedge therefore was rather localized, centered in the pre-midnight region. This finding explains the magnetic field perturbation at GOES 15, which indicated that the spacecraft was located at the eastern side of the current wedge. The concentration of the strong westward electrojets in the pre-midnight region (Additional file 1: Figure S2) during this onset also supports this view. The transient large disturbances in D in the pre-midnight region near the MMS foot point shown in Additional file 1: Figure S2 indicate that a westward traveling surge developed, which was accompanied by a strong upward FAC (Inhester et al. 1981). Therefore, although the entire SCW may have been expanding, which involved a large local time region, the flow and field disturbances at MMS observed between 10:01 and 10:03 UT were likely related to development of an intense current wedge associated with localized enhancements in the flow channel. A number of studies have shown evidence of a localized R1 type current system created around the BBFs (e.g., Nakamura et al. 2001; Liu et al. 2013; Palin et al. 2015).

MMS in the southern hemisphere plasma sheet detected consecutive motion of the plasma boundary: first expansion (outward motion) (events 1–2) and then equatorward motion (events 3–4) during the dipolarizations. These findings are the opposite order of motion of the magnetic disturbances reported near the equatorial flow-braking region, where the Earthward motion of the dipolarization front is followed by tailward propagation of dipolarization (Nakamura et al. 2009). It was shown that the Earthward moving front indeed can result in poleward motion of the front in the near-Earth boundary because of the successively reconnected flux tubes that produce the front (Birn et al. 2013), which may be observed as a front moving outward, with local plasma moving equatorward. Therefore, we may interpret that the two events, (1) and (2), are the near-Earth boundary signatures of flow braking, where the front is produced from successively reconnected field lines with inward plasma motion collapse and results in opposite motion leading to a flux pileup process, as well as to the outward motion of the front, as predicted in the simulation of flow braking. However, the directions of ion drift and current sheet motion were both Earthward/equatorward for the subsequent events (3) and (4), as is the case for a dipolarization front of Earthward convecting plasmas. Yet, the speeds between the plasma and the front do not agree well, particularly for event (3), which is different from the majority of the dipolarization fronts that showed signatures of a tangential discontinuity (Schmid et al. 2011).

Both MMS and Geotail, located near the boundary of the plasma sheet, initially recorded FAC layers with the R2 sense. However, it was still at the higher latitude side of the following R1 current (event 2) and therefore could not be the conventional R2 current, as observed in the BBFs (e.g., Liu et al. 2014). A downward FAC tailward of upward FAC was reported in the magnetic disturbances in the nearly geosynchronous region near the SCW demarcation (Nagai et al. 1987), which is more similar to the R0 current. Based on the results from the multipoint analysis of flows and structures, we suggest that these FACs, R0/R1, are also associated with the vortex development caused by localized flow burst, which has also been predicted in a simulated current system around localized flows in a MHD simulation by Birn and Hesse (2014).

To compare with the observations, we refer to the flow and field perturbations around the simulated reconnection flows from the MHD simulation of near-tail reconnection (Birn et al. 2011). Details of the simulation settings are given by Birn et al. (2011). In this simulation, the onset of near-Earth reconnection (at *X* ~ −20) occurs at about *t* = 90. A localized flow burst near midnight starts to stream Earthward from *t* = 125. The associated disturbances of the flow burst with a dipolarization front and flow vortices surrounding the flow burst reach the near-Earth region (*X* = −10) by *t* = 130 and brake, such that their effects diminish near midnight at *t* = 160, whereas the effects expand azimuthally because of the development of other flow channels, which form a large-scale SCW. This model predicts a total R1 current of 0.58 MA (Table 1, Birn and Hesse 2014), which is comparable to 0.6 MA for this event (Additional file 1: Figure S1). The characteristic magnetic field strength assumed in the simulation, 20 nT, is slightly lower than the lobe field in the midtail observed by Geotail, 25 nT. This difference would indicate that the spatial scale in the observations may be lower (i.e., 1.2 *R*_*E*_), than that assumed in the simulation (i.e., 1.5 *R*_*E*_), which would be associated with a slightly thinner tail current sheet configuration. Also, the simulation sets an inner boundary at *X* = −7.5 *R*_*E*_, and the strong dipolar region extends to *X* = −9 *R*_*E*_, such that flow braking mainly takes place outside the *X* = −9 *R*_*E*_ region, whereas the observations of GOES 15, located near the equator inside 7 *R*_*E*,_ detected FAC disturbances. Once these slight differences in settings are taken into account, the MHD simulations accurately reproduce the overall characteristics of the observed field disturbances at different spacecraft, as discussed below.

**Fig. 5:**
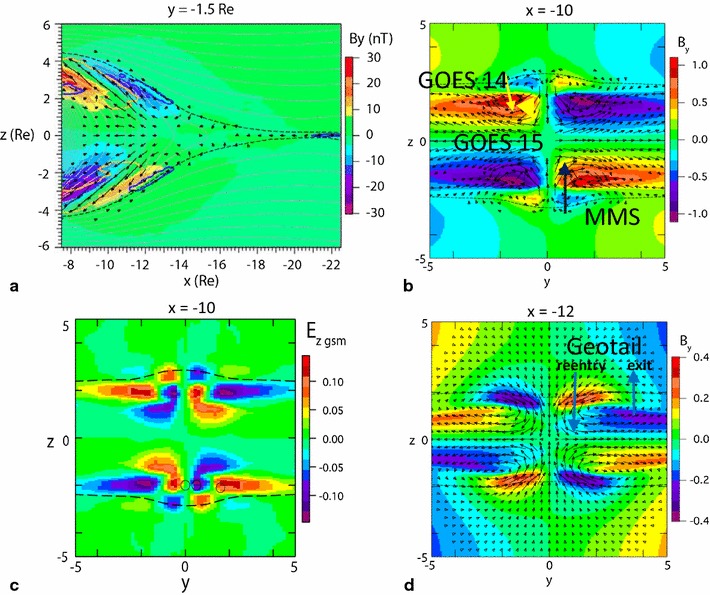
*Ez* and *By* disturbances around the reconnection jets from MHD simulation by Birn and Hesse (2014). **a** Color-coded By in the *X*–*Z* plane at *t* = 132 and *y* = −1.5 together with the current density vectors (Δ*j*) from Birn and Hesse (2014). *Heavy blue* and *orange* contours indicate regions of enhanced tailward and Earthward FACs, respectively. **b** Color-coded By (normalized to 20 nT) in the *Y*–*Z* plane at *t* = 132 and *X* = −10. Possible MMS and GOES 14–15 locations relative to the By pattern during the 10:01 UT dipolarization event (Figs. 3, 4) are indicated by *arrows*. **c** Color-coded vertical (northward) electric field *Ez* (normalized to 20 mV/m) in the *Y*–*Z* plane at *t* = 132 and *X* = −10. **d** Color-coded By in the *Y*–*Z* plane at *t* = 129 at *X* = −12. Possible Geotail location during the 10:14 UT dipolarization event is indicated by an* arrow*. *Black arrows* in **b** and **d** show the *E* × *B* drift vectors

Figure 5a shows color-coded *B*_*Y*_ in the *X*–*Z* plane at *t* = 132 at *y* = −1.5, together with the current density vectors (Δ*j*) from Fig. 8 of Birn and Hesse (2014) during flow braking. Heavy blue and orange contours indicate regions of enhanced tailward and Earthward FACs, respectively. Because this profile is in the dawnside region, the disturbances should correspond to those observed by GOES 14 and 15 in the northern hemisphere (for *Z* > 0). The direction of *B*_*Y*_ disturbances, as well as the FAC direction, should be opposite in the duskside region where MMS (southern hemisphere) and Geotail (northern hemisphere) were located, as indicated by the color-coding in the *Y*–*Z* plane at *t* = 132 and *X* = −10 (Fig. 5b). *B*_*Y*_ and FAC perturbation for a spacecraft entering the flow burst region coincide well with the perturbations seen in the observations. That is, MMS in the southern hemisphere first shows enhancement in negative *B*_*Y*_ (positive *B*_*D*_) perturbation with downward FAC and then positive *B*_*Y*_ (negative *B*_*D*_) perturbation with upward FAC. Geotail in the northern hemisphere also detected *B*_*Y*_ perturbation similar to that shown in Fig. 5a. GOES 14, at higher latitude on the dawnside, observed negative *B*_*Y*_ (positive *B*_*D*_) changes associated with dipolarization, consistent with the motion of the spacecraft inward from the outer boundary. GOES 15, which was located closer to the equator, recorded changes in positive *B*_*Y*_ (negative *B*_*D*_). Possible MMS and GOES 14–15 locations relative to the *B*_*Y*_ pattern during the 10:01 UT dipolarization event (shown in Fig. 3) are indicated by arrows in Fig. 5b. The north–south electric field profile observed by MMS is also consistent with the simulated profile shown in Fig. 5c, which is from the *Y*–*Z* plane at *t* = 132 and *X* = −10. Geotail observed similar changes in *B*_*Y*_, as expected in the northern hemisphere. Figure 5d shows *B*_*Y*_ in the *Y*–*Z* plane as shown in Fig. 5b, except for *t* = 129 and *X* = −12 to represent perturbations around the fast flow tailward of the flow-braking region. The *B*_*Y*_ and *V*_*Y*_ profiles observed during the 10:01 UT and 10:14 UT events correspond to plasma sheet exit and entry, respectively, which can be seen clearly in the simulated pattern.

The observed equatorward electric fields associated with the dawn–dusk magnetic field perturbations and Earthward FAC observed in event (1) have the same polarity as the Hall-field and Earthward FACs carried by inflowing electrons toward the reconnection region tailward of the spacecraft, as previously observed in the lobe/PSBL region (Fujimoto et al. 2001; Nakamura et al. 2004, 2016) and in the outer plasma sheet (Duan et al. 2016) of the near-Earth tail. These current layers are related to thin ion-scale FAC sheets or may contain even smaller scale FAC current sheets (Nakamura et al. 2004, 2016). The field disturbance was observed to propagate faster than the local Alfven speed and was identified as a kinetic Alfvén wave (KAW) disturbance (Duan et al. 2016). In contrast to these signatures, the overall scale of the current sheet of event (1) was relatively large, more than a factor of 10 larger than the ion-gyro scale (i.e., ~70 km), and at least double the ion inertia length, (i.e., ~600 km). Also, we roughly estimate a propagation speed assuming that the disturbance of event (1) is Alfvénic in nature, Δ*E*_*Z*_/Δ*B*_*Y*_ ~ 50 [mV/m]/16 [nT] ~ 3100 km/s, and does not exceed the local Alfvén speed, 5900 km/s. Thus, the observed large-scale current sheet structures, such as events (1) and (2), associated with the dawn–dusk flows shown in this study are less likely to be explained by a propagating Hall-field disturbance via KAW.

In the MHD simulation (Birn and Hesse 2014), the downward current resulting from flows toward midnight is a part of the vortices generated outside of an Earthward flow burst, but does not reach the ionosphere (and therefore was not considered part of the current wedge loops). The observed FACs (event *i*) shown in Fig. 3 are mainly carried by upward-flowing electrons. An interesting consequence of the observed downward FAC at the boundary (event *i*) being a part of the BBF-related vortices is that a part of the BBF Earthward propagating energy is intended for the acceleration process of electrons within the magnetosphere. Alternatively, the observed downward FAC may also correspond to the R0 current observed in the ionosphere at the high-latitude side of the auroral bulge (Fujii et al. 1994; Gjerloev and Hoffman 2002). Our observations then suggest that the R0 type current may also be driven by the flow vortex in the flow-braking region.

Although the overall dawn–dusk deflection of the flow and field is interpreted as the localized BBF and associated MHD scale process as above, we note that the role of small-scale disturbances is also significant during these events. For example, there are multiple peaks seen in the *E* × *B* velocity profile in addition to the overall changes in ion flows. In addition, a sharp peak in the electric field and magnetic field is seen near the end of event (1). It should also be noted that current sheets (3) and (4), which occur after the large-scale flow disturbances, are very intense and have spatial scales comparable to ion scales. Therefore, multiscale processes may determine the dynamics of the plasma sheet boundary. Details on these multiscale properties of the current sheet, including the kinetic-structures, are subject to separate studies.

## Summary and conclusion

Multiscale observations by MMS in the southern hemisphere plasma sheet recorded consecutive motion of plasma sheet expansion and equatorward motion during the multiple dipolarizations and enhanced plasma flows in the plasma sheet boundary in the flow-braking region. This motion enabled MMS to cross the FAC layers with a distinct pattern (upward/downward FAC located equatorward/poleward, respectively, accompanied by duskward/dawnward *V*_*y*_ flows and outward/inward *E*_*z*_). Such a pattern is consistent with those expected in the southern hemisphere duskside of a localized (3D effect) BBF, as reported in Birn and Hesse (2014). Geotail in the duskside northern hemisphere, as well as GOES 14–15, located dawnside in the northern hemisphere, shows evidence supporting this interpretation. These observations demonstrate that multipoint observations monitoring the motion of the current sheet simultaneously with the current direction are essential to understand the evolution of the FAC near the flow-braking region.

## Additional file

**Additional file 1. Figure S1.** Local time distribution of the disturbances in H(X) and D(Y) components of mid-latitude magnetic fields at 10:00 UT, August 10, 2016. The *red profiles* show the data, while the *blue dots* show the results from the SCW model (Sergeev et al. 2011). The mid-latitude ground-based magnetic field disturbances shown in the plots are used to deduce the magnetic local time distribution and the total current of the SCW. **Figure S2.** **a** H and **b** D components of the nightside high-latitude magnetogram disturbances. The *vertical lines* show the 09:42 and 09:57 UT onset.

## Abbreviations

BBF: bursty bulk flow
CPS: central plasma sheet
EIS: energetic ion spectrometer
FAC: field-aligned current
FPI: Fast Plasma Instruments
GOES: Geostationary Operational Environmental Satellite
MMS: Magnetospheric Multiscale
PSBL: plasma sheet boundary layer
SCW: substorm current wedge
