# Supplementary material for: Near-Earth plasma sheet boundary dynamics during substorm dipolarization
Source: Earth Planets Space. 2017 Sep 18;69(1):129. doi: 10.1186/s40623-017-0707-2 (PMC6961498; doi:10.1186/s40623-017-0707-2)
Supplement: Supplementary file 1 — Additional file 1. Figure S1. Local time distribution of the disturbances in H(X) and D(Y) components of mid-latitude magnetic fields at 10:00 UT, August 10, 2016. The red profiles show the data, while the blue dots show the results from the SCW model (Sergeev et al. 2011). The mid-latitude ground-based magnetic field disturbances shown in the plots are used to deduce the magnetic local time distribution and the total current of the SCW. Figure S2. a H and b D components of the nightside high-latitude magnetogram disturbances. The vertical lines show the 09:42 and 09:57 UT onset. [file 40623_2017_707_MOESM1_ESM.pdf]

SUPPLEMENT FIGURES

Figure S1. Local time distribution of the disturbances in H(X) and D(Y) components of mid-latitude magnetic fields at 10:00 UT, August 10, 2016. The red profiles show the data, while the blue dots show the results from the SCW model [Sergeev et al., 2011]. The mid-latitude ground-based magnetic field disturbances shown in the plots are used to deduce the magnetic local time distribution and the total current of the SCW.

Figure S2. H and D components of the nightside high-latitude magnetogram disturbances. The vertical lines show the 09:42 and 09:57 UT onset.

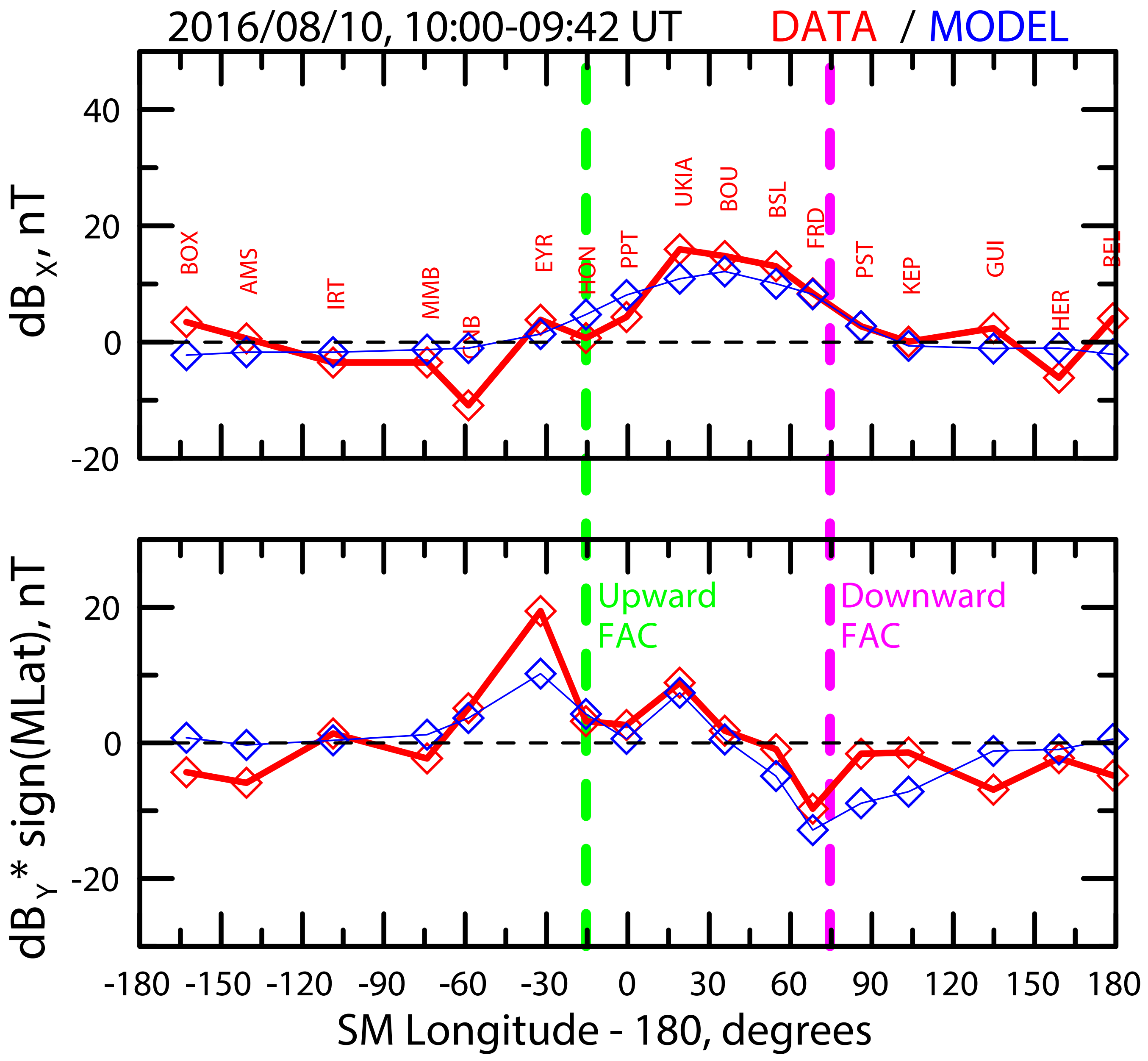
2016/08/10, 10:00-09:42 UT
DATA / MODEL
dBX', nT
BOX
AMS
IRT
MMB
EYR
HON
PPT
UKIA
BOU
BSL
FRD
PST
KEP
GUI
HER
Upward
FAC
Downward
FAC
dBY * sign(MLat), nT
40
20
0
-20
-180
-150
-120
-90
-60
-30
0
30
60
90
120
150
180
SM Longitude - 180, degrees

Figure S1

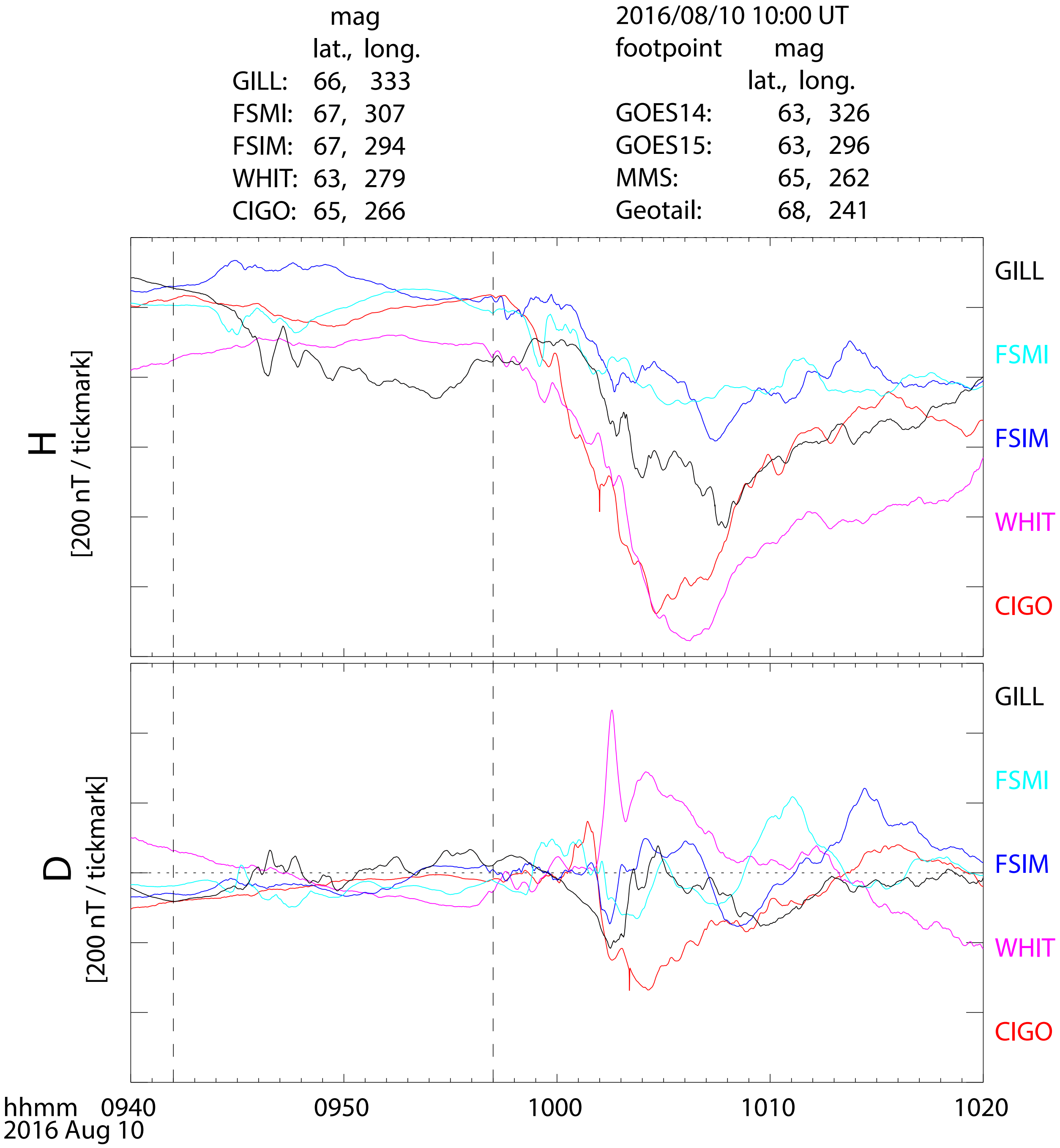
mag
lat., long.
GILL: 66, 333
FSMI: 67, 307
FSIM: 67, 294
WHIT: 63, 279
CIGO: 65, 266
2016/08/10 10:00 UT
footpoint mag
lat., long.
GOES14: 63, 326
GOES15: 63, 296
MMS: 65, 262
Geotail: 68, 241
H
[200 nT / tickmark]
GILL
FSMI
FSIM
WHIT
CIGO
D
[200 nT / tickmark]
GILL
FSMI
FSIM
WHIT
CIGO
hhmm 0940
2016 Aug 10
0950
1000
1010
1020

FIGURE S2
